# Controlled and cellulose eco-friendly synthesis and characterization of Bi_2_O_2_CO_3_ quantum dot nanostructures (QDNSs) and drug delivery study

**DOI:** 10.1038/s41598-020-78266-6

**Published:** 2020-12-04

**Authors:** Hojat Samarehfekri, Hamid Reza Rahimi, Mehdi Ranjbar

**Affiliations:** 1 Student Research Committee, Kerman University of Medical Sciences, Kerman, Iran; 2 Neuroscience Research Center, Institute of Neuropharmacology, Kerman University of Medical Sciences, Kerman, Iran; 3 Department of Pharmacology and Toxicology, Faculty of Pharmacy, Kerman University of Medical Sciences, Kerman, Iran; 4 Pharmaceutics Research Center, Institute of Neuropharmacology, Kerman University of Medical Sciences, P.O. Box: 76175-493, 76169-11319 Kerman, Iran

**Keywords:** Biochemistry, Chemical biology, Drug discovery, Medical research, Chemistry, Materials science, Nanoscience and technology

## Abstract

This work aimed to prepare solvent-free or green Bi_2_O_2_CO_3_ for quantum dot nanostructures (QDNSs) based on cellulose as a stabilizer and green capping agent to sorafenib delivery for liver targeting. Because the walnut tree is one of the most abundant trees in Iran, it was tried to synthesize Bi_2_O_2_CO_3_ QDNSs using a walnut skin extract. The saturation magnetization for Bi_2_O_2_CO_3_ QDNSs was calculated to be 68.1. Also, the size of products was measured at around 60–80 nm with the Debye–Scherrer equation. Moreover, the morphology, functional groups, and crystallography of the Bi_2_O_2_CO_3_ nanoparticles were investigated using atomic force microscopy, scanning electron microscopy, vibrating-sample magnetometer, and Uv–vis spectroscopy. The results demonstrated that Bi_2_O_2_CO_3_ QDNSs have opto-magnetic properties and they can be suggested as the candidate materials for the sorafenib delivery on the liver tissue. The optical band gap estimated for Bi_2_O_2_CO_3_ QDNSs was found to be red-shift from 3.22 eV. This study suggests the preparation of the Bi_2_O_2_CO_3_ QDNSs based on cellulose as new opto-magnetic materials at different temperatures of 180 °C, 200 °C, 220 °C, and 240 °C for sorafenib delivery as a type of biological therapy drug.

## Introduction

In the past three decades^1–3^, nanotechnology has seen significant advances in nanomaterials and new methods and materials. The liver is the hub of the metabolic activity of the body. With the advances in nanopharmaceutical sciences^4,5^, more goals have been pursued in service delivery structures to reduce the side effects of different drugs^6^, as well as improving drug delivery^7,8^ in economically feasible ways^9^. Nanoparticle-based anti-cancer drugs have improved the treatment of various liver diseases and different types of cancer with drug targeting and imaging agents for liver cancer imaging. In particular, magnetic and optic Bi_2_O_2_CO_3_ NPs-based delivery systems provide a good-looking method for localizing sorafenib as clinically important oral tyrosine kinase drugs in the liver using magnetic forces and optical effects. These drugs act at relatively long-range and do not affect most biological tissues. Sorafenib is an inhibitor for the treatment of various cancers^10^. With the development of new materials or methods, concern about environmental pollution has been doubled due to nanoparticles produced by chemical methods and the production of dangerous side-effects for in vivo preclinical imaging and drug discovery^11–13^. Therefore, there is a need for clean, non-toxic, and environmentally friendly green chemistry^14^. There are different physical and chemical methods for the synthesis of nanostructures in medicine such as chemical remediation^15,16^, lithography^17^, the ultrasonic method^18^, co-precipitation^19^, and microwave method^20^. The disadvantages of this method are the use of chemicals that act as restorative and stabilizing agents^21^. Nevertheless, these materials cause environmental pollution and these methods have a low production rate in high pressure and high energy during the reaction process^22^. Solvent-free synthesis as an eco-friendly useful approach^23^ helps synthesize nano compounds^24^ without using chemical and dangerous solvents^25^. The skin green walnut contains 13 phenolic compounds. Among these compounds, joggle is the most frequent and is the main ingredient in the green skin of walnut^26^. The thermal decomposition method for synthesizing monodispersed metal nanoparticles is one of the best ways to control particle sizes and regular-shaped and well-crystallized nanocrystals^27^. Today, imaging of living organisms is one of the ways to intercept drugs to treat cancer cells and tumors in the body of living organisms^28^. Optical drug tracking is performed using various materials that have a high potential in optical properties^29^. Due to the opto-magnetic properties of nanoparticles, recent studies on the case using the nanostructures as career drugs have been expanded rapidly Table 1.

**Table 1:** Recent research on the case using the nanostructures for synthesis of nanoparticles and nanostructures.

Types	Purpose	Synthesis	Size	References
Fe_3_O_4_/polymer	Degradation	Self-assembly	80–120 nm	^30^
Co NPs	Photodynamic	Templated	≤ 30 nm	^31^
CdSe/Fe_3_O_4_-chitosan	In vivo imaging	Thioglycolic acid	60 nm	^32^
SiO_2_–e_2_O_3_	Agent delivery	Coprecipitation	40–60 nm	^32^
Co-doped ZnO NPs	Antibacterial	Microwave combustion method	≤ 50 nm	^33^

Because of their unique properties such as large surface to volume ratio and the quantum confinement effect^34^, nanoparticles exhibit corresponding changes in the emission properties^35^. The cellulose crystalline structures (CCS) act not only as a suitable substrate for the reductant agent of Bi_2_O_2_CO_3_ QDNSs but also serves as the green capping agent and packaging factor. In this study, we report a new chemical processing without using any solvent (solvent-free method) for the synthesis of Bi_2_O_2_CO_3_ QDNSs. This is a novel and useful opto-magnetic nanomaterial for sorafenib delivery for liver targeting in in-vivo imaging studies. The connection of Bi_2_O_2_CO_3_ QDNSs to sorafenib provides opto-magnetic routing. The synthesized products were characterized by X-ray diffraction (XRD), energy dispersive spectroscopy (EDX), Atomic force microscopy (AFM), scanning electron microscopy (SEM), transmission electron microscopy (TEM), and UV–vis spectroscopy.

## Results and discussion

**Figure 1:**
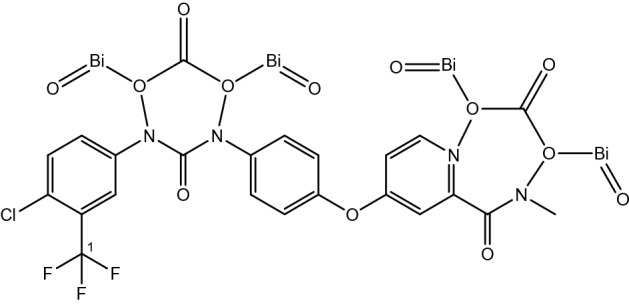
The suggested Bi_2_O_2_CO_3_/Sorafenib formulation structures.

These Bi_2_O_2_CO_3_ QDNSs with opto-magnetic properties will enhance drug bioavailability and reduce dosing frequency and prevent hard and impossible treatment therapy. The structures of the suggested Bi_2_O_2_CO_3_/sorafenib formulation are illustrated in Fig. 1.

### Crystallography

**Figure 2:**
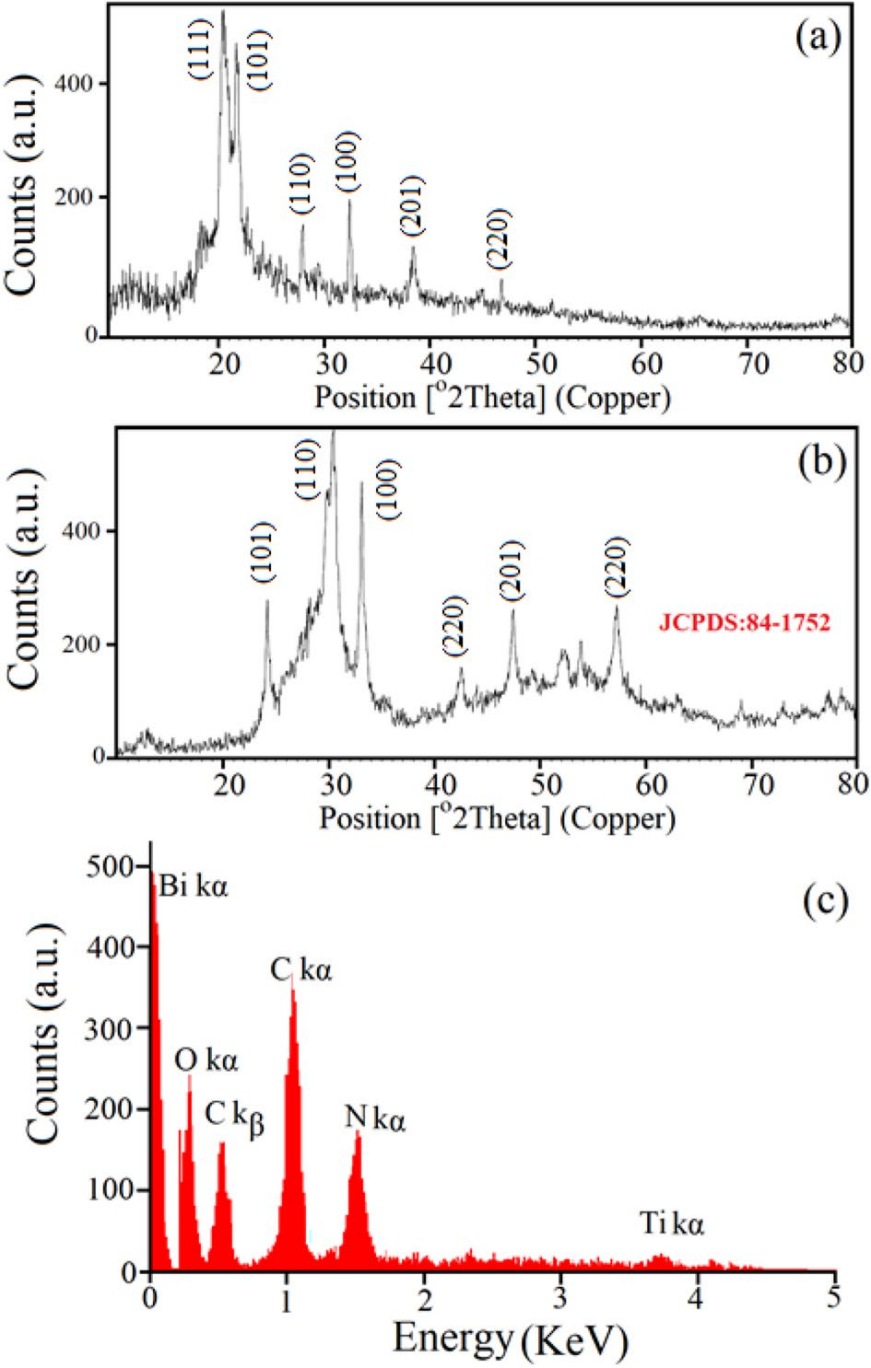
XRD pattern of the cellulose nanocrystals (**a**), Bi_2_O_2_CO_3_ QDNSs in the cellulose base (**b**) and EDAX spectrum of the as synthesized Bi_2_O_2_CO_3_ QDNSs (**c**).

To investigate the crystallographic properties and crystalline phases, the products were measured with X-ray diffraction (XRD) throughout the manufacturing process. The XRD patterns (10 < 2θ < 80) of cellulose nanocrystals and Bi_2_O_2_CO_3_ nanocrystals in the cellulose base are shown in Fig. 2a,b, respectively. Using the XRD results, most of the reflection peaks can be indexed according to tetragonal phases with crystallographic parameters a = 5.468, b = 27.32, and c = 5.46 through the following lattice parameter formula (Eq. 1).

1 $$ a = d\surd \left( {h^{2} + \, k^{2} + \, l^{2} } \right) $$

where h, k, and l are the crystalline face indexes. The specific surface area (S_a_) of the Bi_2_O_2_CO_3_ nanocrystals was obtained from the maximum intensity peak (112) while X-ray density (D_x_) was calculated using Eqs. (2) and (3).

2 $$ {\text{S}}_{{\text{a}}} = 6/{\text{DD}}_{{\text{x}}} $$

3 $$ {\text{D}}_{{\text{x}}} = 8{\text{M}}/{\text{N}}_{{\text{a}}} {\text{a}}^{3} $$

where N_a_ is Avogadro number (6.023 × 1023 atoms/mole), M is molar weight, and a is the lattice parameter. Crystallography data show that nanoparticles are likely smaller if the nucleation rate is larger than the crystal growth rate. The results show that the obtained products lack any impurities. Based on the XRD data, the crystalline size and diameter (Dc) of Bi_2_O_2_CO_3_ nanoparticles are calculated about 30–80. These values are estimated using the Scherer equation (Eq. 4) from the diffraction patterns from the full width of the half maximum (FWHM):

4 $$ {\text{D}} = K\lambda /\beta \;\cos \;\uptheta $$

where D is the mean size of crystallites (nm), K is crystallite shape factor with approximate amount 0.9, λ is the X-ray wavelength, β is FWHM in radians of the X-ray diffraction peak, and θ is the Braggs angle (°).

### Morphological properties

EDX analysis is considered as a non-destructive analytical technique used to identify the percentage of elemental components. A typical EDX spectrum of the Bi_2_O_2_CO_3_ QDNSs is represented in Fig. 2c. Here, bismuth molecule (Bi) peaks indicate the existence of Bi elements in Bi_2_O_2_CO_3_ QDNSs, the presence of elements such as C and O elements related to cellulose molecule, and very small quantities of N elements related to the existence of NO_3_ in precursors. The presence of very small amounts of Ti element can be attributed to unpredictable impurities in the reaction condition and measurement environment.

**Figure 3:**
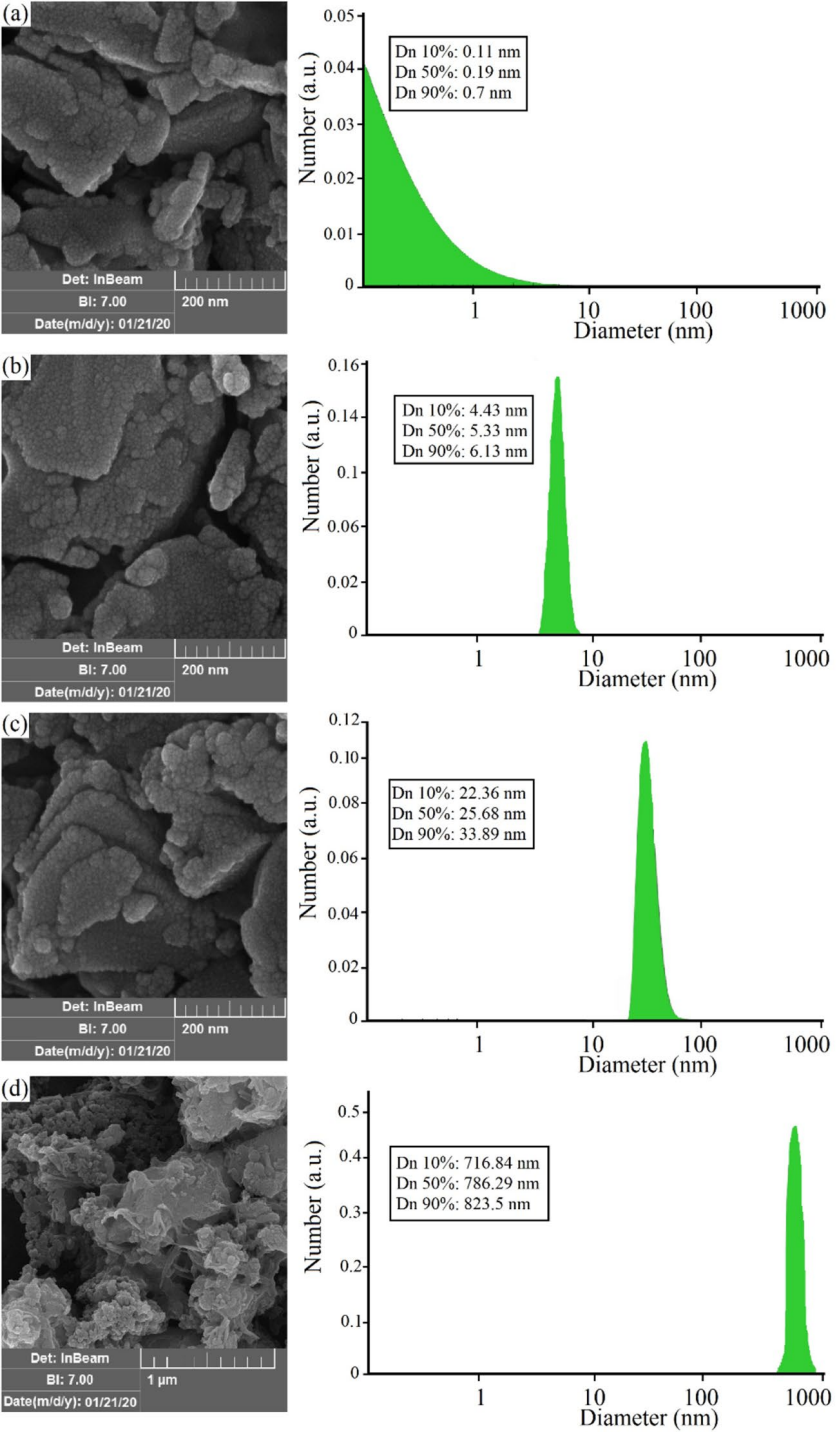
SEM images of the Bi_2_O_2_CO_3_ QDNSs at 180 °C (**a**), 200 °C (**b**), 220 °C (**c**) and 240 °C for 4 h (**d**).

Figure 3a–d represent SEM images of Bi_2_O_2_CO_3_ QDNSs treated at 180 °C, 200 °C, 220 °C, and 240 °C, respectively, for 4 h. Based on the SEM images, at 180 °C, Bi_2_O_2_CO_3_ QDNSs show scale-like structures in the presence of the cellulose molecules. As can be seen, with increasing temperature, the density of scale-like structures will increase in the samples. As the temperature gradually rises to 200 °C, the Bi_2_O_2_CO_3_ QDNSs grow on the cellulose surface and a large number of sheet structures can be found in the SEM images. In some areas, there is an overlap between the sheets. Cellulose molecules can play the role of the reductant agent and as a carbon source. Also, they can be responsible for control size and shape capping agent effects in the synthesis process of Bi_2_O_2_CO_3_ QDNSs. Aggregation of nanoparticles occurs due to excessive nucleation at high temperatures. As a result, activation of the surfaces of the nanoparticles causes the product made from Bi_2_O_2_CO_3_ QDNSs to have a larger particle size. It can be observed that Bi_2_O_2_CO_3_ QDNSs synthesized at 220 °C on cellulose-based have more uniform size and shape and these samples have smaller particles size than other samples.

As temperature rises from 220 °C to 240 °C, the SEM image indicates an increase in the particle size of the products and generation of bulk Bi_2_O_2_CO_3_ QDNSs on the cellulose substrate through the thermal decomposition method. In this process, due to the high surface area of the Bi_2_O_2_CO_3_ QDNSs, the particle surfaces aggregated together and bulky structures are achieved when exposed to more heating.

### Structural properties

**Figure 4:**
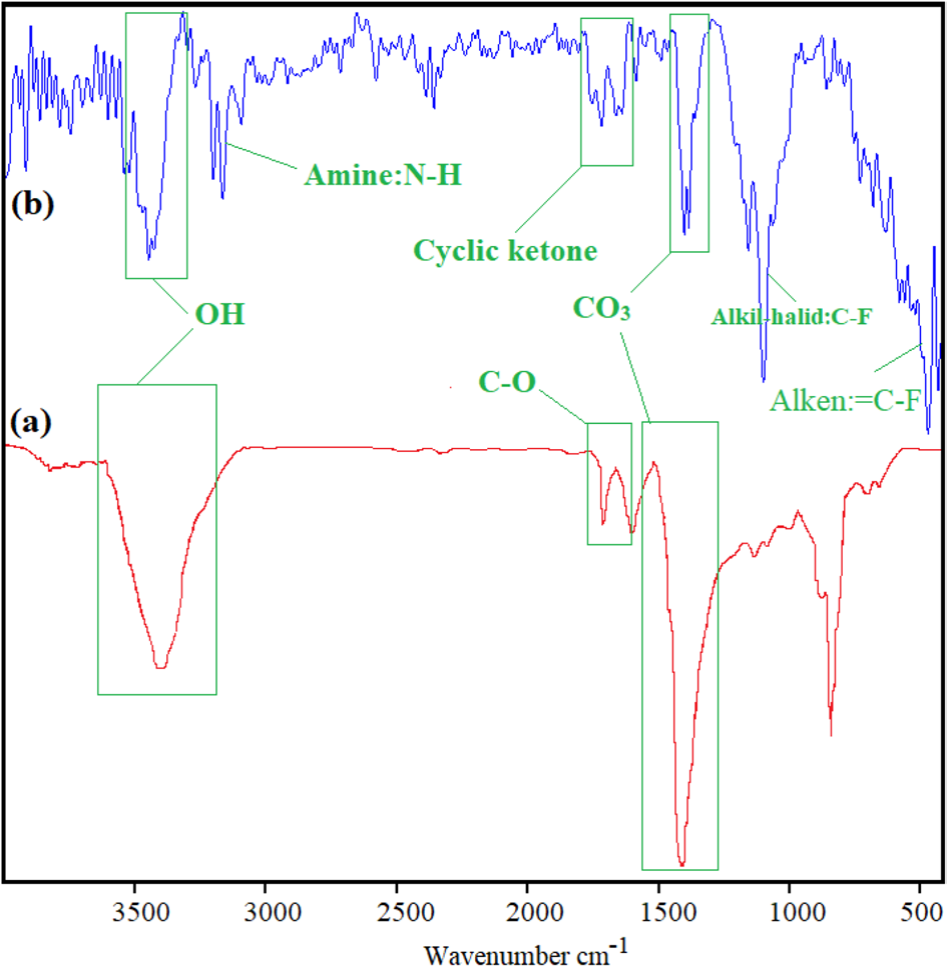
FT-IR of Bi_2_O_2_CO_3_ QDNSs (**a**) and Bi_2_O_2_CO_3_/Sorafenib formulation (**b**).

Fourier transform infrared (FT-IR) spectrum is among the most important, non-destructive, and emerging tools that are widely used to identify organic (and in some cases inorganic) and functional groups in materials. FT-IR of Bi_2_O_2_CO_3_ QDNSs and Bi_2_O_2_CO_3_/sorafenib formulation are shown in Fig. 4a,b, respectively. The broad absorption band centered at 1400–1450 cm^−1^ and 850 cm^−1^ correspond to the antisymmetric stretching mode of the CO_3_ group in Bi_2_O_2_CO_3_ nanostructures^36^. The absorption band appeared in the area 3300–3400 cm^−1^ is related to amine (N–H) bands in Bi_2_O_2_CO_3_/sorafenib formulation^37^. Two strong bands at 675 cm^−1^ and 1230 cm^−1^ with different intensities are respectively attributed to stretching of the alkene=C–F and Alkyl-Halide–C–F bonds in sorafenib molecular structures.

### Surface properties

**Figure 5:**
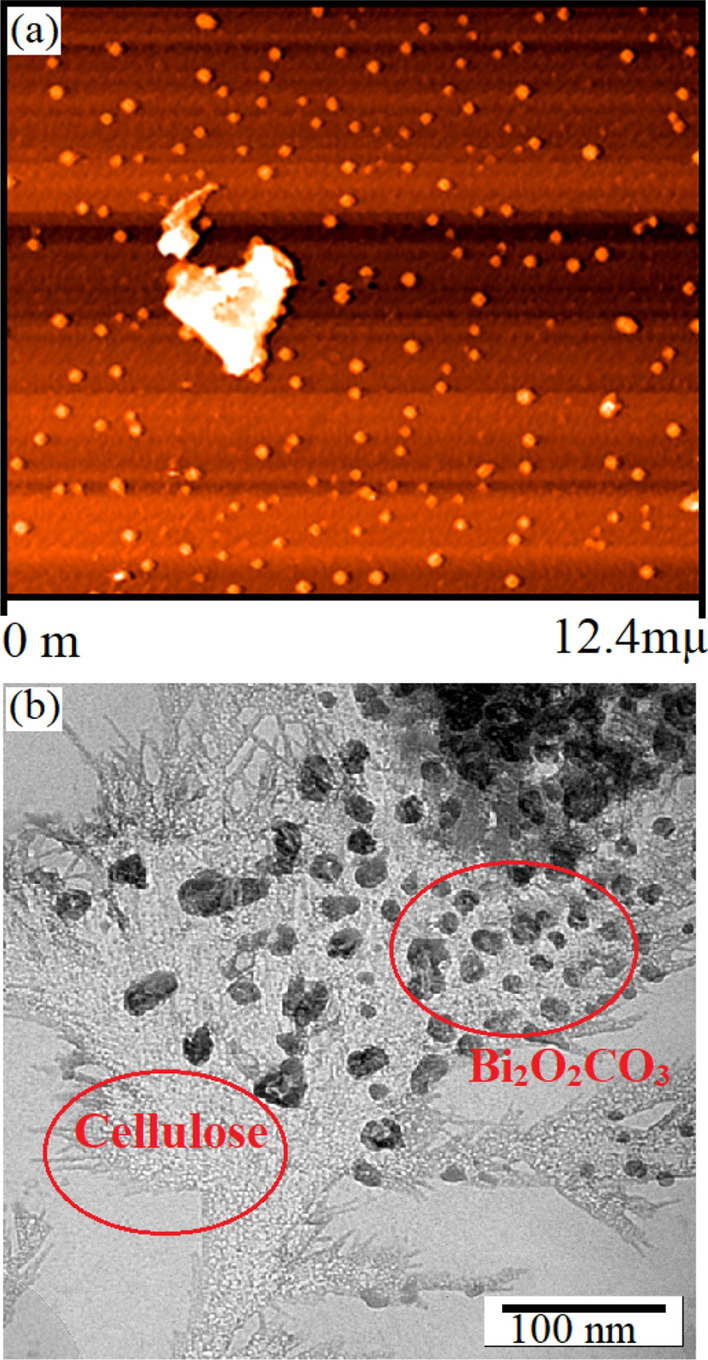
AFM images of Bi_2_O_2_CO_3_ QDNSs based on the cellulose surface composites 180 °C at 4 h (**a**) and TEM image the final nanostructures (**b**).

AFM as a non-destructive analysis provides topography of the surface of the samples. AFM images of the Bi_2_O_2_CO_3_ QDNSs based on the cellulose surface composites (sample c) are shown in Fig. 5a. The results show that the Bi_2_O_2_CO_3_ nanoparticles are distributed uniformly on the cellulose surface, leading to properties such as opto-magnetic performance. The root-mean-squared roughness (RMS) is an important parameter to introduce the roughness and softness and surface properties of the products. According to the AFM image data, R_rms_ as the standard deviation factor is determined as follows (Eq. 5):

5 $$ R_{rms } = \sqrt {\frac{{\mathop \sum \nolimits_{{{\text{n}} = 1}}^{{\text{N}}} ({\text{z}}_{{\text{n}}} - {{\check{\text{z}}}})^{2} }}{N - 1}} $$

where z_n_ denotes the height of the *n*th data, *ž* shows the mean height of Z_n_ in AFM topography, and N represents the number of data. For an area of 87.43 pm^2^, the roughness average is 14.62 nm. To assess the morphology and distribution size crystal of the product in the optimum condition (sample c), TEM images were studied (Fig. 5b). It can be observed from the TEM image that the Bi_2_O_2_CO_3_ QDNSs (sample c) have uniform size and shape as the cellulose molecules appear in the range of 15–40 nm. The quantitative determination of the solutions, ions, and efficiency conjugated drug compounds were calculated using the Uv–vis spectroscopy.

**Figure 6:**
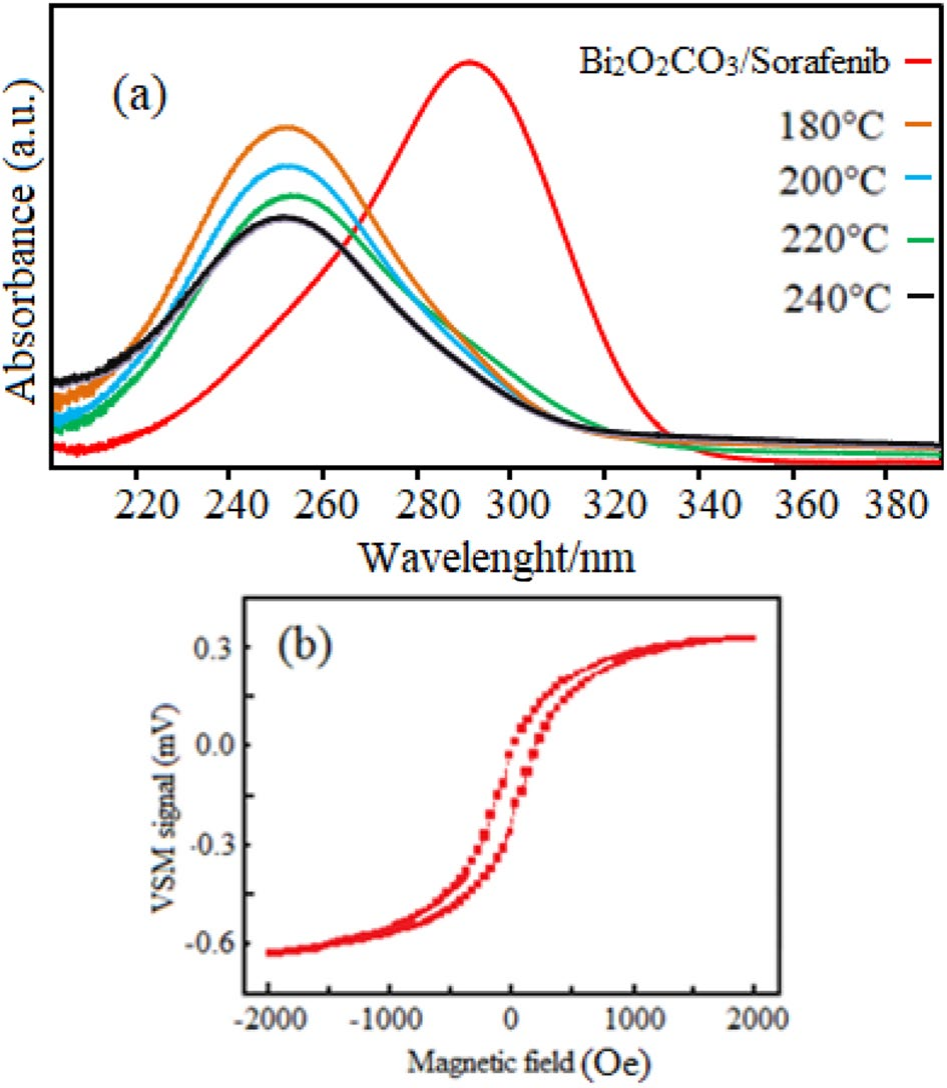
UV–vis spectra of the as synthesized Bi_2_O_2_CO_3_ QDNSs based on the cellulose in different temperatures such as 180 °C, 200 °C, 220 °C, 240 °C (**a**), hysteresis loop of the Bi_2_O_2_CO_3_/Sorafenib formulation in air (**b**).

The magnetic properties of the samples were measured with a vibrating sample magnetometer (VSM). The Uv–vis spectroscopies of Bi_2_O_2_CO_3_ QDNSs in different temperature conditions and Bi_2_O_2_CO_3_/sorafenib formulation samples are illustrated in Fig. 6a. The emission peaks at 252 nm in Bi_2_O_2_CO_3_ nanostructures correspond to the exciton recombination. The emission peaks at 292 nm in Bi_2_O_2_CO_3_ NPs/sorafenib formulation are related to oxygen deficiency and lattice distortion molecular interactions of Bi_2_O_2_CO_3_ nanostructures and sorafenib. Magnetic properties of the nanoparticles’ diamagnetic, paramagnetism, ferromagnets, antiferromagnets, and ferrimagnets materials are measured using the VSM. Figure 6b indicates the magnetization curve of the Bi_2_O_2_CO_3_/Sorafenib formulation in the air. Using magnetic properties in nanoparticles can create trackable and stable properties in various drugs and cause their interactions with cells or biological proteins, leading to the increased compatibility of drugs. The area within a loop represented in the hysteresis curve indicates magnetic energy loss. The B–H magnetic hysteresis curve for the Bi_2_O_2_CO_3_/sorafenib formulation indicates a very small hysteresis behavior for the samples. It also exhibits small values of coercivity field and remnant magnetization. The saturated magnetization (Bs) values and for Bi_2_O_2_CO_3_ nanoparticles/sorafenib formulation at a magnetic field of 1.5 T was obtained to be 68.1 emu/g. The Bi_2_O_2_CO_3_ nanostructure shows an obvious ferromagnetic behavior, suggesting the presence of the Bi molecule that can exist in the spinel systems.

### Bi_2_O_2_CO_3_/sorafenib formulation delivery

**Figure 7:**
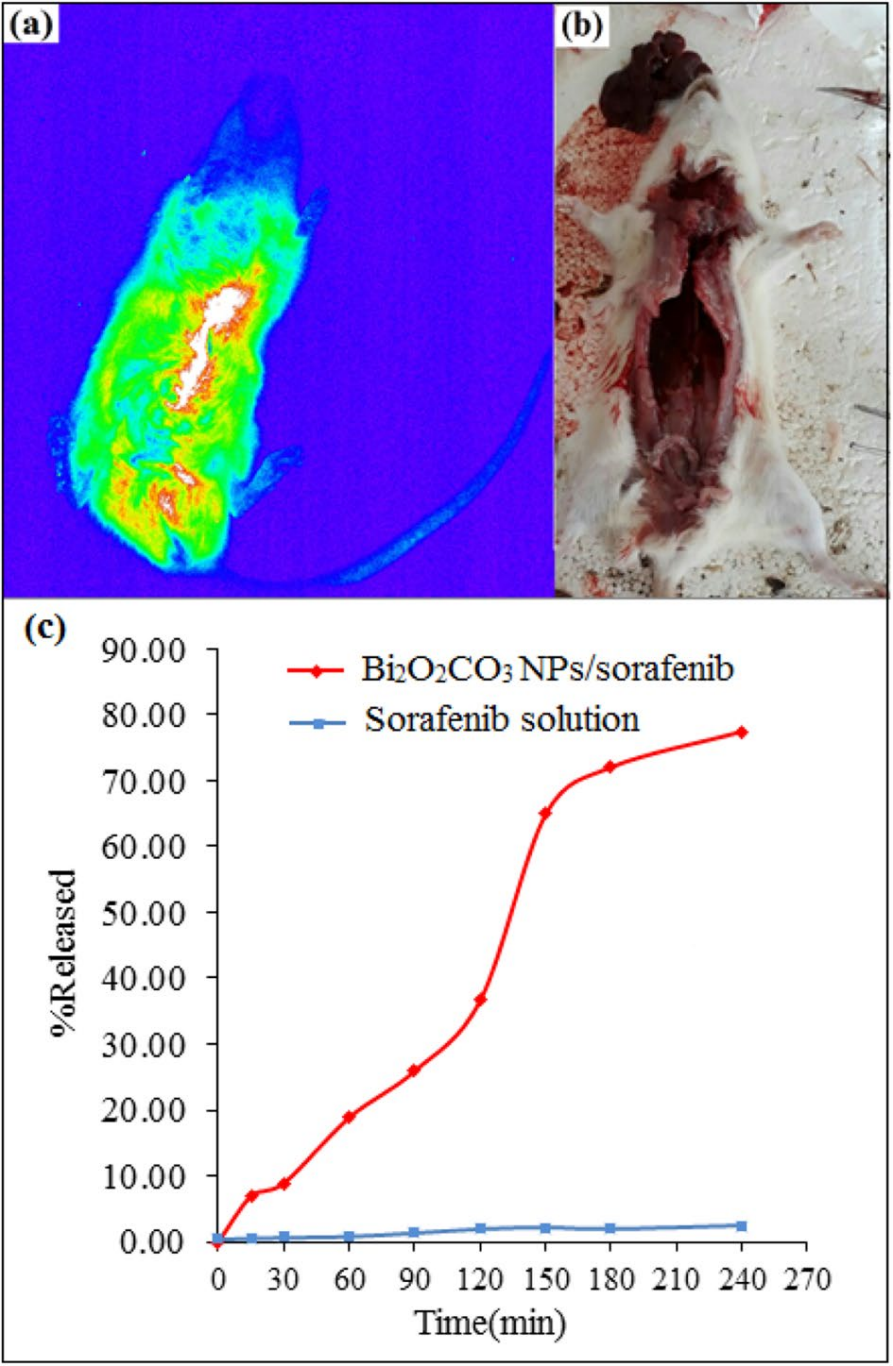
Image of liver mice 2 min after of injection (**a**) and the same rat after two months later for the phenotypic investigation (**b**), the cumulative release profiles of Bi_2_O_2_CO_3_/sorafenib formulation structures and Bi_2_O_2_CO_3_ QDNSs (**c**).

Initially, no optical properties were observed, but after 2 min, the image shows that the nano substance also contains the drug inside the liver. The evaluation showed that the synthesized material did not have a fatal effect. So, all organs were evaluated phenotypically to ensure that no damage was done to the target tissue and other organs. There was no change in the body tissues, especially the liver tissue. Therefore, it is suggested testing the levels of assurance of designs with different concentrations of Bi_2_O_2_CO_3_ nanoparticles/sorafenib in a large number of in rates species, along with phenotypic and gene expression tests. The liver image of mice 2 min after injection and the same rate after 2 months later for the phenotypic investigation are illustrated in Fig. 7a,b, respectively. In cancer cells, glucose consumption is several times. Cancer cells can be detected by conjugated Bi_2_O_2_CO_3_ QDNSs to glucose structures and tracking these structures in the body. Vertical all-glass Franz-type diffusion cell with an active surface area of 2.37 cm^2^ and a receptor phase volume of 15 ml was used for the release study of sorafenib from Bi_2_O_2_CO_3_ QDNSs at 37 ± 1 °C. Cellulose acetate dialysis membrane (Visking tube, MW cut-off 12,000 D) was used as a barrier between donor and receptor compartments of the diffusion cell. The receptor compartment was filled with normal saline solution (0.9%) and the donor compartment section was filled with 1 ml Bi_2_O_2_CO_3_ nanoparticles/sorafenib. Free drug solutions were used as controls sample and empty Bi_2_O_2_CO_3_ structures were examined as blanks. For this purpose, 1 ml sample was withdrawn at fixed time intervals from the receptor compartment, the same amount of fresh receptor medium was replaced, and the permeated drug concentration was measured. The cumulative release profiles of Bi_2_O_2_CO_3_/sorafenib formulation structures and Bi_2_O_2_CO_3_NPs are presented in Fig. 7c. The results indicate that after 240 min, 77.51% sorafenib was released from Bi_2_O_2_CO_3_/sorafenib formulation structures, while the amount of the drug released for sorafenib solution was 2.47%. The reason is that, due to the high surface area of the nanostructures, they absorb more amount drugs on their surface.

## Conclusions

This study demonstrated that the Bi_2_O_2_CO_3_ QDNSs can act as an optical and magnetical delivery platform for the tracing sorafenib by magnetic and optical targeting. In the present study, Bi_2_O_2_CO_3_ QDNSs were synthesized and characterized as optical-magnetical carriers for sorafenib delivery in the living organism in the mice. For this purpose, BALB/c male mice were purchased from the Animal Care Center of Research Center, Institute of Neuropharmacology, Kerman University of Medical Sciences, Kerman, Iran. We propose Bi_2_O_2_CO_3_ QDNSs as green chemistry with significant optical and magnetic properties that can be used in the in vivo imaging of the living organism and also in optical density drugs. The TEM image proves that the size of the Bi_2_O_2_CO_3_ QDNSs (sample c) structures are in the range of 15–40 nm. After 240 min, 77.51% and 2.47% sorafenib was released from Bi_2_O_2_CO_3_/sorafenib and sorafenib solution formulation respectively.

## Material and methods

### Chemicals and equipment

All the chemical and material reagents used in this study were used with no further purification as they were of pure and analytical grade. The starting reagent Bi(NO_3_)_3_ was purchased from SRL Company, India, and walnut shells were prepared from gardens around the Sirjan City of Iran. Sorafenib drug type was prepared from Loghman pharmaceutical and hygienic Co. The devices used in this study are as follows: X-ray diffractometer using Ni-filtered Cu-Ka radiation by a Rigaku D-max C III was used for XRD analysis. The EDS analysis was studied using the XL30 Philips microscope. For the morphological properties, SEM devices (JXA-8100, and JSM-6700F) were used. TEM (images were captured using a TEM Philips EM208 device with an accelerating voltage of 200 kV in the University of Shahid Bahonar Kerman, Iran. The FTIR spectrometer (550 Nicolet in KBr pellets) was recorded in the range of 400–4000 cm^−1^. AFM for surface morphology study was done using an easy scan 2 Advanced Research AFM device. The particle size and particle distribution of the nanoparticles were determined by laser-light scattering (Mastersizer 2000E, Malvern Instruments, UK). In vivo imaging was obtained using in vivo imaging system F Pro model Kodak manufacturing company in the USA. The UV–visible spectrum of the product was recorded using a Scinco UV–vis scanning spectrometer (ModelS-4100).

### Synthesis of cellulose nanocrystals

Initially, 1 g of dry walnut skin without any dust and moisture was used as the starting reagent. Then, the precursor was put into the external boat box-like and transferred to the furnace. The furnace temperature was set to 350 °C and the samples were heated for 8 h. After the heating process, the system was allowed to cool to room temperature naturally. The black precipitations were characterized by SEM and XRD.

### Synthesis of Bi_2_O_2_CO_3_ nanocrystals

In the next step, 0.4 g Bi(NO_3_)_3_ powder was completely ground in a porcelain mortar. Then, the powder was transferred to a crucible containing black brown precipitations cellulose crystals from the previous step. All these experiments were performed in the same way. The samples were heated in the furnace at different temperament conditions including 180 °C, 200 °C, 220 °C, and 240 °C for 4 h. After the thermal treatment in each step, the system was allowed to cool under argon atmosphere to room temperature naturally and the as-synthesized final precipitations were collected and were characterized by SEM, XRD, Uv–vis, and FT-IR.

### Bi_2_O_2_CO_3_/sorafenib formulation

To prepare Bi_2_O_2_CO_3_/sorafenib formulation, 50 mg/l of as-synthesized Bi_2_O_2_CO_3_ nanocrystals was solved in 20 ml distilled water. Also, 30 mg/l of sorafenib powder was solved in 10 ml of distilled water and ethanol solution with the ratio 2:1 and then was added dropwise to the above solution. The pH was adjusted between 6 and 7.5 with a digital pH meter. Next, the mixture was placed under the reflux process in vigorous stirring for 2 h using a magnetic stirrer at room temperature. Finally, for homogeneity, the suspension was placed under the ultrasonic bath in 60 watts for 15 min.

### Animals and treatment

All the experimental procedures were carried out according to the protocols set for working with animals and for the care and use of laboratory animals at Kerman University of Medical Sciences (Kerman, Iran). Males Balb/c with an approximate weight of about 150–200 g were fed with a standard diet. They were kept under 12:12 h light/dark cycles, at 20 °C, and relative humidity of 25–30% at an animal farm with the local ethics code of 97000588 from the Kerman University of Medical Sciences. The anesthesia was done using Ketamine/xylazine with a ratio of 1:2 followed by immediate injection of Bi_2_O_2_CO_3_/sorafenib formulation to the rat tail (Balb/c male).

### Ethical approval

This article does not contain any studies with human subjects. The mice (BALB/c male purchased from Animal care center) were feeded and raised according to the Institutional Animal Care and Use Committee (IACUC) protocol.

## References

[1] Zhong W (2009). Nanomaterials in fluorescence-based biosensing. Anal. Bioanal. Chem..

[2] Giannitrapani L (2014). Nanotechnology applications for the therapy of liver fibrosis. World J. Gastroenterol..

[3] Turkez H (2014). The risk evaluation of tungsten oxide nanoparticles in cultured rat liver cells for its safe applications in nanotechnology. Braz. Arch. Biol. Technol..

[4] Kim K (2012). High fat diet-induced gut microbiota exacerbates inflammation and obesity in mice via the TLR4 signaling pathway. PLoS ONE.

[5] Rim P (2011). pH-tunable calcium phosphate covered mesoporous silica nanocontainers for intracellular controlled release of guest drugs. Angew. Chem. Int. Ed..

[6] Zhang J, Ellsworth K, Ma PX (2010). Hydrophobic pharmaceuticals mediated self-assembly of β-cyclodextrin containing hydrophilic copolymers: novel chemical responsive nano-vehicles for drug delivery. J. Control. Release.

[7] Florence A (2018). Nanotechnologies for site specific drug delivery: changing the narrative. Int. J. Pharm..

[8] Ahmed M, Ghanem A (2014). Chiral β-cyclodextrin functionalized polymer monolith for the direct enantioselective reversed phase nano liquid chromatographic separation of racemic pharmaceuticals. J. Chromatogr. A.

[9] Chen H, Yada R (2011). Nanotechnologies in agriculture: new tools for sustainable development. Trends Food Sci. Technol..

[10] Lin T (2016). Development and characterization of sorafenib-loaded PLGA nanoparticles for the systemic treatment of liver fibrosis. J. Control. Release.

[11] Campos G (2018). In vitro and in vivo experimental models employed in the discovery and development of antiepileptic drugs for pharmacoresistant epilepsy. Epilepsy Res..

[12] Gunn N, Rabiner A (2017). Imaging in central nervous system Drug discovery. Semin. Nucl. Med..

[13] Tournier N, Stieger B, Langer O (2018). Imaging techniques to study drug transporter function in vivo. Pharmacol. Ther..

[14] Baskin M (2007). Copper-free click chemistry for dynamic in vivo imaging. Proc. Natl. Acad. Sci..

[15] Okuo J (2018). Synthesis characterization and application of starch stabilized zerovalent iron nanoparticles in the remediation of Pb-acid battery soil. Environ. Nanotechnol. Monit. Manag..

[16] Tsakiroglou D (2018). A numerical model to simulate the NAPL source zone remediation by injecting zero-valent iron nanoparticles. Chem. Eng. Sci..

[17] Park J (2018). Fabrication of Al_2_O_3_ nano-micro patterns by Al_2_O_3_ dispersion resin using UV imprint lithography. Thin Solid Films.

[18] Ayanda S, Nelana M, Naidoo E (2018). Ultrasonic degradation of aqueous phenolsulfonphthalein (PSP) in the presence of nano-Fe/H2O2. Ultrason. Sonochem..

[19] Prosapio V, Marco D, Reverchon E (2018). Supercritical antisolvent coprecipitation mechanisms. J. Supercrit. Fluids.

[20] Mondal S (2018). Rapid microwave-assisted synthesis of gold loaded hydroxyapatite collagen nano-bio materials for drug delivery and tissue engineering application. Ceram. Int..

[21] Jayaseelan C (2013). Biological approach to synthesize TiO_2_ nanoparticles using Aeromonas hydrophila and its antibacterial activity. Spectrochim. Acta Part A Mol. Biomol. Spectrosc..

[22] Leung DY, Wu X, Leung M (2010). A review on biodiesel production using catalyzed transesterification. Appl. Energy.

[23] Azeredo J (2014). A solvent-and metal-free synthesis of 3-chacogenyl-indoles employing DMSO/I2 as an eco-friendly catalytic oxidation system. J. Organ. Chem..

[24] Li H (2016). Eco-friendly and rapid microwave synthesis of green fluorescent graphitic carbon nitride quantum dots for vitro bioimaging. Sens. Actuat. B Chem..

[25] Kakati D, Sarma J (2011). Microwave assisted solvent free synthesis of 1, 3-diphenylpropenones. Chem. Cent. J..

[26] Reza MM (2016). Green synthesis of NiFe2O4/Fe2O3/CeO2 nanocomposite in a Walnut Green Hulls extract medium: magnetic properties and characterization. Curr. Nanosci..

[27] Blázquez G (2012). Copper biosorption by pine cone shell and thermal decomposition study of the exhausted biosorbent. J. Ind. Eng. Chem..

[28] Pu K (2014). Semiconducting polymer nanoparticles as photoacoustic molecular imaging probes in living mice. Nat. Nanotechnol..

[29] Jiang S, Gnanasammandhan M, Zhang Y (2010). Optical imaging-guided cancer therapy with fluorescent nanoparticles. J. R. Soc. Interface.

[30] Du P (2013). Biocompatible magnetic and molecular dual-targeting polyelectrolyte hybrid hollow microspheres for controlled drug release. Mol. Pharm..

[31] Kim S (2007). Organically modified silica nanoparticles co-encapsulating photosensitizing drug and aggregation-enhanced two-photon absorbing fluorescent dye aggregates for two-photon photodynamic therapy. J. Am. Chem. Soc..

[32] Liu L (2012). Preparation of magnetic and fluorescent bifunctional chitosan nanoparticles for optical determination of copper ion. Microchim. Acta.

[33] Basith NM (2014). Co-doped ZnO nanoparticles: structural, morphological, optical, magnetic and antibacterial studies. J. Mater. Sci. Technol..

[34] Harris LK, Theriot JA (2018). Surface area to volume ratio: a natural variable for bacterial morphogenesis. Trends Microbiol..

[35] Zhang Y (2019). Quantum confinement luminescence of trigonal cesium lead bromide quantum dots. Appl. Surf. Sci..

[36] Huang H (2014). Syntheses, characterization and nonlinear optical properties of a bismuth subcarbonate Bi_2_O_2_CO_3_. Solid State Sci..

[37] Mian F (2017). Bi_12_O_17_Cl_2_/(BiO)_2_CO_3_ nanocomposite materials for pollutant adsorption and degradation: modulation of the functional properties by composition tailoring. ACS Omega..

